# Modelling of ciprofloxacin killing enhanced by hyperbaric oxygen treatment in *Pseudomonas aeruginosa* PAO1 biofilms

**DOI:** 10.1371/journal.pone.0198909

**Published:** 2018-06-14

**Authors:** Peter Alexander Vistar Gade, Terkel Bo Olsen, Peter Østrup Jensen, Mette Kolpen, Niels Høiby, Kaj-Åge Henneberg, Thomas Sams

**Affiliations:** 1 Biomedical Engineering, Dept. of Electrical Engineering, Technical University of Denmark, DK-2800 Lyngby, Denmark; 2 Dept. of Applied Mathematics and Computer Science, Technical University of Denmark, DK-2800 Lyngby, Denmark; 3 Department of Clinical Microbiology, Rigshospitalet, DK-2100 Copenhagen, Denmark; 4 Costerton Biofilm Center, Department of Immunology and Microbiology, Faculty of Health and Medical Sciences, University of Copenhagen, DK-2200 Copenhagen, Denmark; Laurentian, CANADA

## Abstract

**Outline:**

In chronic lung infections by *Pseudomonas aeruginosa* (PA) the bacteria thrive in biofilm structures protected from the immune system of the host and from antibiotic treatment. Increasing evidence suggests that the susceptibility of the bacteria to antibiotic treatment can be significantly enhanced by hyperbaric oxygen treatment. The aim of this study is to simulate the effect of ciprofloxacin treatment in a PAO1 biofilm model with aggregates in agarose when combined with hyperbaric oxygen treatment. This is achieved in a reaction-diffusion model that describes the combined effect of ciprofloxacin diffusion, oxygen diffusion and depletion, bacterial growth and killing, and adaptation of the bacteria to ciprofloxacin. In the model, the oxygen diffusion and depletion use a set of parameters derived from experimental results presented in this work. The part of the model describing ciprofloxacin killing uses parameter values from the literature in combination with our estimates (Jacobs, et al., 2016; Grillon, et al., 2016). Micro-respirometry experiments were conducted to determine the oxygen consumption in the *P. aeruginosa* strain PAO1. The parameters were validated against existing data from an HBOT experiment by Kolpen *et al.* (2017). The complete oxygen model comprises a reaction-diffusion equation describing the oxygen consumption by using a Michaelis-Menten reaction term. The oxygen model performed well in predicting oxygen concentrations in both time and depth into the biofilm. At 2.8 bar pure oxygen pressure, HBOT increases the penetration depth of oxygen into the biofilm almost by a of factor 4 in agreement with the scaling that follows from the stationary balance between the consumption term and diffusion term.

**Conclusion:**

In the full reaction-diffusion model we see that hyperbaric oxygen treatment significantly increases the killing by ciprofloxacin in a PAO1 biofilm in alignment with the experimental results from Kolpen *et al.* (Kolpen, et al., 2017; Kolpen, et al. 2016). The enhanced killing, in turn, lowers the oxygen consumption in the outer layers of the biofilm, and leads to even deeper penetration of oxygen into the biofilm.

## Introduction

*Pseudomonas aeruginosa* is a gram-negative bacterium associated with the lung disease cystic fibrosis (CF) [5]. CF is an autosomal recessive disease caused by a defect in the cystic fibrosis transmembrane conductance regulator gene (*CFTR*) [6]. CFTR is a chloride ion channel that reduces the mucus volume of the epithelial lining fluid leading to dehydration and mucociliary dysfunction [7, 8]. The stagnation of mucus contributes to accumulation of bacteria, including *Pseudomonas aeruginosa*. Consequently, a sustained inflammatory response results in a gradual decline in lung function ultimately causing death for CF patients [9, 10].

Microscopical examinations indicate that *P. aeruginosa* resides in microcolonies, also known as biofilms, surrounded by numerous polymorphonuclear leukocytes (PMN) in the endobronchial mucus in lungs from CF patients with chronic lung infection [11]. *In vivo* measurements have revealed intense depletion of oxygen (O_2_) in the endobronchial mucus in CF patients with chronic lung infection [12]. The extreme depletion of O_2_ is reproduced in newly expectorated endobronchial secretions as steep oxyclines [13, 14] and is predominately caused by O_2_ consumption for production of reactive O_2_ and nitrogen species by the PMNs [15–17]. Consequently, O_2_ consumption by aerobic respiration is very small and anaerobic bacterial respiration is favoured in the O_2_ depleted parts of endobronchial secretions from CF patients [13, 18]. The ability of the O_2_ consumption by the accumulated PMNs to restrict *P. aeruginosa* is further evidenced by the inhibition of aerobic growth of *P. aeruginosa* by the PMNs in the endobronchial mucus in CF lungs [19].

Studies suggest that O_2_ limitation in the adhered biofilm resulting in low metabolic activity is correlated with high survival rates of *P. aeruginosa*’s during antibiotic treatment [20]. The biofilms in CF lungs are, however, not adhered to solid surfaces, but are embedded as small aggregates in the viscous endobronchial mucus [21]. To mimic the organisation of *P. aeruginosa* in small aggregates surrounded by viscous mucus we have embedded *P. aeruginosa* in agarose allowing us to confirm that the killing of *P. aeruginosa* biofilm by ciprofloxacin is oxygen dependent and to demonstrate that hyperbaric oxygen treatment (HBOT) increases the killing of *P. aeruginosa* by ciprofloxacin [4]. To further approach the situation in the infected CF lungs we mimicked the PMN-mediated low availability of oxygen for aerobic bacterial respiration by culturing the biofilms embedded in agarose for three days before assessing the effect of HBOT on the killing of *P. aeruginosa* during treatment with ciprofloxacin [3]. In this way we could demonstrate that when aerobic respiration was enabled by reoxygenation during HBOT, *P. aeruginosa* became susceptible to ciprofloxacin, thus confirming the contribution of bacterial respiration to increased bacterial killing by antibiotics [22]. *P. aeruginosa* is known to have five terminal oxidases in the electron transport chain which directly consume oxygen by reduction [23]. Although less efficient, *P. aeruginosa* is also capable of anaerobic respiration with N-oxides as terminal electron acceptors, leaving its overall metabolism complex [24, 25].

CF patients are treated with a wide range of antibiotics [26, 27]. The killing effect of some antibiotics, e. g. fluoroquinolones, aminoglycosides, and beta-lactams is enhanced under aerobic conditions due to formation of reactive oxygen species (ROS) [4, 28], hence oxygen availability and aerobic respiration are relevant for treatment. Two antibiotics used to treat *P. aeruginosa* biofilm infections in CF are tobramycin and ciprofloxacin [26]. Tobramycin has shown a considerable time delay for the penetration into biofilms which probably increases the adaptive response in the bacteria [29]. Several simulation studies of the bacterial killing of *P. aeruginosa* with and without adaptation have been carried out with various antibiotics, including ciprofloxacin, illuminating their pharmacokinetic and dynamic properties on *P. aeruginosa* infection and how time kill-curves evolve during treatment [30–32]. However, none have yet linked the observed increased killing from HBOT to an antibiotic adaptation model.

**Fig 1:**
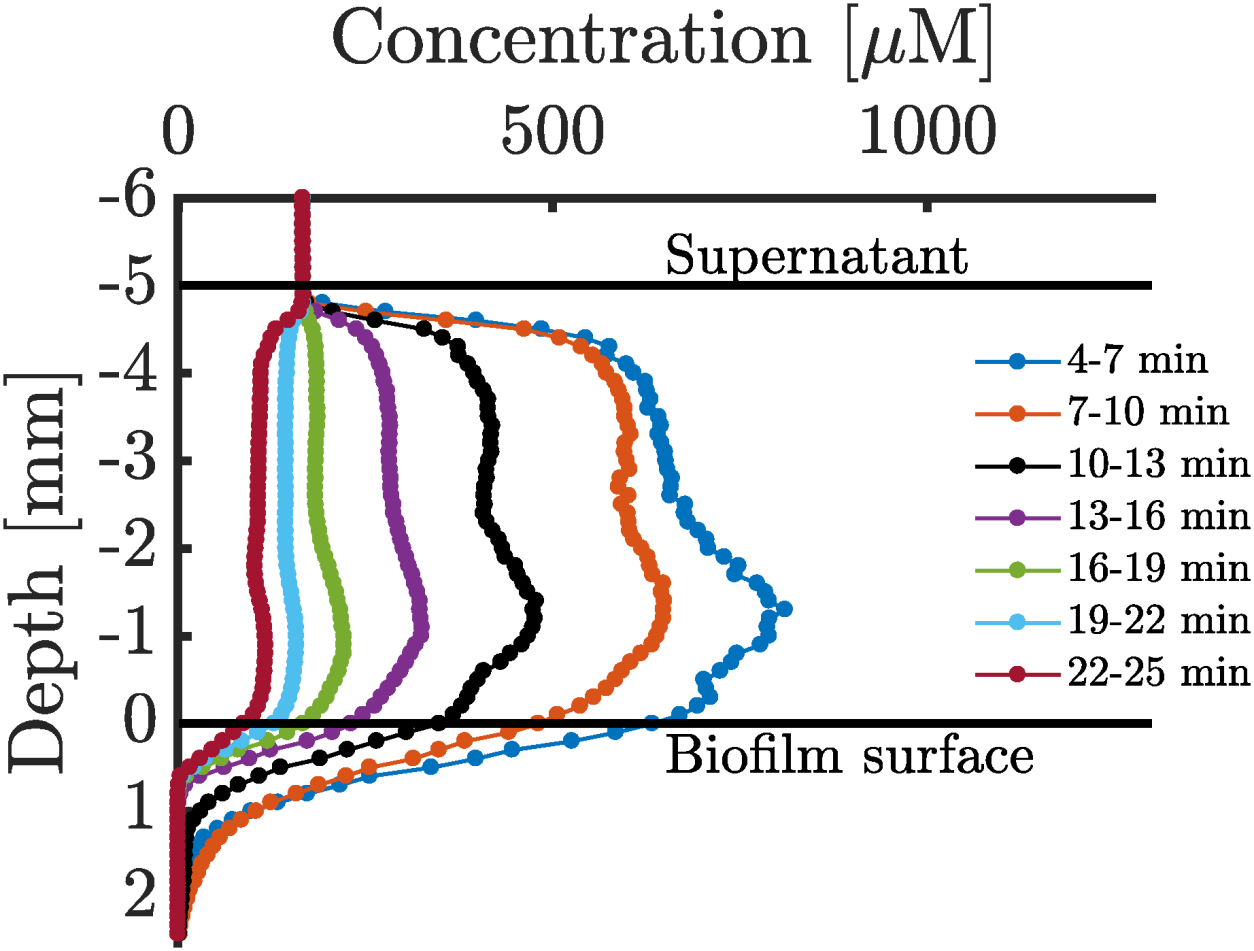
Oxygen profiles sampled after 90 minutes of HBOT. Oxygen profiles are recorded after 90 minutes of HBOT applied to a 5 mm thick agarose biofilm, hence an unloading process of an already oxygen penetrated biofilm is observed. Oxygen profiling was initiated 4 minutes after the 90 minutes HBOT and every profile takes approximately 3 minutes to record. Horizontal black bars represent supernatant and biofilm surfaces, respectively. The concentration of oxygen in the supernatant immediately after treatment is approximately 1000 *μ*M as the chamber has to be decompressed to 1 atm. The biofilm is present for depth 0 mm to 5 mm while the supernatant is present in the region from −5 mm to 0 mm. The supernatant is displayed primarily to verify that the mixing is strong in this region. Figure modified from [3].

This paper investigates the dynamics of O_2_ treatment and the resulting O_2_ concentration profiles in a biofilm model of *P. aeruginosa*. The model parameters are determined in respirometry experiments and from the O_2_ penetration and unloading dynamics reported by Kolpen *et al.* [3] and shown in Fig 1. Using the oxygen model in combination with a modified version of an existing antibiotic model with ciprofloxacin treatment by Jacobs *et al.* (2016) [1] and Gregoire *et al.* (2010) [30], the dynamics of bacterial killing in a biofilm is investigated and the limitations of this and existing models are discussed.

## Materials and methods

### Bacterial strain and growth media

The standard laboratory *P. aeruginosa* strain PAO1 was obtained from Stover *et al.* [33]. Growth was established in Lysogeny broth (LB) [5 g/L yeast extract (Oxoid, Basingstoke, UK), 10 g/L tryptone (Oxoid) and 10g/L NaCl (Merck, Rahway, NJ), pH 7.5], incubated overnight at 37°C and shaken at 150 rpm. Overnight cultures were used in micro-respirometry experiments to estimate O_2_ consumption in stationary cultures. For O_2_ consumption experiments in exponential cultures, the overnight culture was diluted in LB to OD_600_ = 0.01 and grown until OD_600_ = 0.4. Bacterial CFU counts were determined on solid lactose agar plates (‘Blue plates’ based on a modified Conradi–Drigalski medium containing 10 g/L detergent, 1 g/L Na_2_S_2_O_3_ ⋅ H_2_O, 0.1 g/L bromothymolblue, 9 g/L lactose and 0.4 g/L glucose, pH 8.0; Statens Serum Institut, Copenhagen, Denmark) to select for Gram-negative bacteria. All plates were incubated overnight at 37°C.

### Oxygen depletion measurements

For bulk O_2_ depletion experiments in planktonic cultures respiration vials (OXVIAL4) with integrated stripes of the O_2_ sensitive REDFLASH-indicators glued to the inner wall (Pyro-Science, GmbH) were positioned on top of a magnetic stirring head connected to a magnetic stirrer (Model 300, Rank Brothers, Cambridge, UK). The O_2_ concentration measurements were recorded with separately available adaptor rings (ADVIAL4, Pyro-Science) to allow easy fixation of bare optical fibers (SPFIB-BARE, Pyro-Science) which connect the respiration vials to an optical oxygen meter (FireStingO_2_, FSO2-4, Pyro Science). The software Pure Oxygen Logger (v.3.206 with FireStingO_2_ (Firmware 3.07), Pyro-Science) measured O2 in continuous mode with sampling every 1 s. The used channels were activated; the respective sensor code, output unit, as well as environmental conditions for the sample temperature ([°C]), pressure ([mbar]), and salinity ([g/L]) were entered. The temperature was fixed at 37°C and the pressure was fixed at 1013 mbar. The factory calibration was used to calibrate the sensors (OXVIAL4) according to the manufacturer’s recommendation. O_2_ concentrations were measured in both stationary and exponential cultures diluted 10 times in LB.

For vertical space-time profiles in biofilms we used the data reported by Kolpen *et al.* [3]. For completeness, we provide brief experimental details for these data: PAO1 biofilms were grown and treated under anoxic conditions in an anaerobic growth chamber. The optical density at 600 nm (OD_600_) of overnight cultures of PAO1 was adjusted to 0.4 before 100-fold dilution in LB medium supplemented with 2% 2-hydroxyethyl-agarose (Sigma-Aldrich, Brøndby, Denmark) and 1 mL was loaded into 24-well microtiter plates to achieve a cell loading of ∼10^6^ cells/ml. The biofilm size was 5.7 mm high and 15 mm wide. The medium was supplemented with anoxic NaNO_3_ (1 mM) to enable anaerobic respiration. The supernatant was replaced daily with 1 mL of LB medium supplemented with 1 mM NaNO3. Microtiter plates were covered with Parafilm and lid and were incubated under anoxic conditions at 37°C for 3 days. The 3 day old biofilm was treated for 90 min with HBOT (100% O_2_ at 2.8 bar). Less than 1 minute after ending the treatment, the microtiter plate was positioned on a heated metal rack and kept at 37°C. The vertical O_2_ profiles were recorded with a fiber-optic O_2_ microsensor positioned with a micromanipulator (50 *μ*m tip diameter, FireSting2, Profix version 4.51; Pyro-Science GmbH, Germany). The microsensor was calibrated according to the manufacturer’s recommendations (air saturated and O_2_-free water). The microsensor was positioned at the base of the biofilm sample and profiles were recorded while moving the sensor in vertical steps of 100 *μ*m through the biofilm sample.

## Analysis

Overview of Analysis: First we model the oxygen consumption in a well mixed culture as described in “Reaction model”. This serves to determine the basic parameters needed to describe the oxygen consumption. Next, in “Reaction-diffusion model for oxygen”, we combine the obtained oxygen consumption model with a simple diffusion model. This allows us to verify that the hyperbaric oxygen penetration into a biofilm model reported by [3] and shown in Fig 1 are qualitatively described. Finally, in “Ciprofloxacin model with oxygen consumption”, a full model describing the killing by ciprofloxacin in combination with hyperbaric oxygen treatment is constructed. This model includes a mechanism for reversible adaptive oxygen-dependent killing. This enables us to give predictions for the combined effect of HBOT and ciprofloxacin treatment.

### Reaction model

A simple reaction model using a Michaelis-Menten reaction term is presented in Eq (1) where *c* is the molar concentration of oxygen. The model includes two parameters, namely *K*_*m*_ and *R*_max_. The latter is the maximum consumption rate and has in other studies [34–37] been decomposed into a subset of parameters as seen in Eq (2), where *μ*_max_ is the maximum specific growth rate with units of h^−1^, *ε*_*c*_ is the dimensionless volume fraction occupied by cells, *ρ*_*x*_ is the intrinsic cellular oxidase density with units *μ*M, and *Y*_*x*,*o*_ is the dimensionless oxygen yield coefficient. Importantly, Eq (1) reveals that the overall consumption in a biofilm scales linearly in the volume fraction occupied by cells.

$$\begin{array}{c} \frac{dc}{dt}=-R_{\text{max}}\frac{c}{K_{m}+c} \end{array}$$ (1)

$$\begin{array}{c} R_{\text{max}}=\frac{\mu_{\text{max}}\;\varepsilon_{c}\;\rho_{x}}{Y_{x,o}} \end{array}$$ (2)

The parameter *K*_*m*_ acts as a cut-off value, indicating the oxygen concentration below which limitation sets in. Even though PA has five terminal oxidases which reduce oxygen [24], we shall describe the process using just one value for *K*_*m*_. This works well, since the individual cutoffs all lie within an order of magnitude from *K*_*m*_ = 4 *μ*M [23]. Our maximal oxygen reaction velocity therefore represents the sum of contributions from all oxidases.

The solution to Eq (1) is obtained by integration as presented in Eq (3) where *c*(0) is the initial concentration of oxygen.

$$\begin{array}{c} c\left(t\right)=K_{m}\;\text{W}\;\left(\frac{c(0)}{K_{m}}\;\exp\left(\;\frac{c\left(0\right)-R_{\text{max}}\;t}{K_{m}}\;\right)\;\right) \end{array}$$ (3)

**Fig 2:**
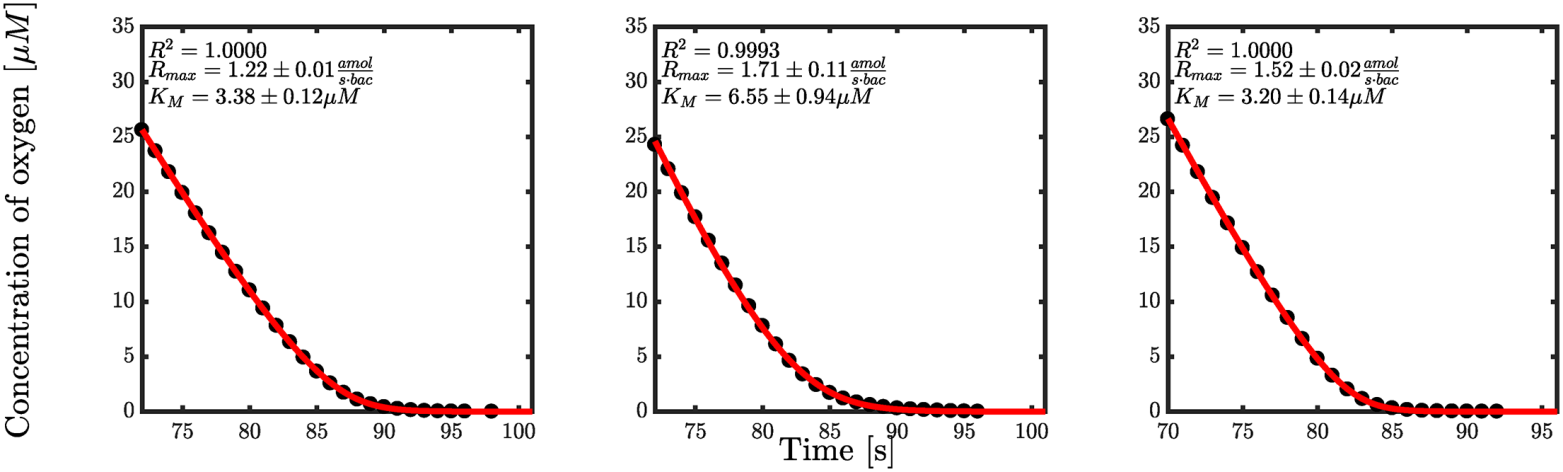
Oxygen consumption for overnight cultures of PAO1. Dots represent experimental data and the red lines are the solution from Eq (3) with respective parameters.

The solution involves the Lambert W function, which is the inverse of *x* → *x*e^*x*^ [38]. Estimates of *R*_max_ and *K*_*m*_ may now be established by fitting the experimental data as illustrated in Fig 2. Experimental data is obtained from respirometry experiments as described in Materials and Methods.

In Fig 2, three typical respirometry profiles and fits with Eq (3) are shown. Using the solution from Eq (3), the value of *K*_*m*_ was determined from a total of 14 respirometry experiments shown in S1–S4 Figs. The parameter values providing the best fit were computed using the Levenberg-Marquardt algorithm in MATLAB with an integer weighting to compensate for the statistical underrepresentation of data in the tail.

The average *K*_*m*_ for the first batch of experiments with six similar cultures is 3.4 *μ*M (see S1 and S2 Figs). In Fig 2, three of these are shown. Additionally, another batch of eight respirometry experiments were conducted where an average *K*_*m*_ of 0.62 *μ*M was observed (see S3 and S4 Figs). Clearly, *K*_*m*_ is very low and the bacteria can thus easily be saturated with oxygen. The variation in *K*_*m*_ probably arises from the interactivity of the five terminal oxidases present in *P. aeruginosa* [39]. A study by Arai *et al.* [23] determined *K*_*m*_ for each oxidase to be between 0.23-4.3 *μ*M corresponding well to the respirometry results.

The estimate of *R*_max_ in Fig 2 is centered around 1.5 attomol per second per bacteria but some variation of this parameter was seen across the 14 different profiles. The mean value of *R*_max_ was calculated to be $2.57\;\frac{\text{amol}}{\text{s}\cdot \text{bac}}$ and ranges from a minimum value of $1.22\;\frac{\text{amol}}{\text{s}\cdot \text{bac}}$ to a maximum value of $5.70\;\frac{\text{amol}}{\text{s}\cdot \text{bac}}$. Interestingly, it was observed that the cultures that exhibited exponential growth had an average *R*_max_ value of $4.48\;\frac{\text{amol}}{\text{s}\cdot \text{bac}}$ which would indicate a shift in consumption rate per bacteria when entering a stationary phase. However, the number of profiles of exponentially growing cultures was low (*n* = 3) and further respirometry experiments are therefore required to confirm this.

### Reaction-diffusion model for oxygen

As a first attempt to understand the effect of HBOT in biofilm, oxygen profiles were sampled immediately after HBOT by Kolpen *et al.* (2017) in [3] as reproduced in Fig 1. In the experiment, a series of oxygen profiles were sampled after 90 minutes of HBOT as the oxygen level returned to normoxic. By modelling the oxygen profiles one should be able to predict the condition of the biofilm during treatment. Assuming that the underlying oxygen consumption mechanism is similar to that in the planktonic cells in the respirometry experiments, the data can be described by adding a diffusion term to the rhs of Eq (1). We then get a 1-dimensional reaction-diffusion equation as seen in Eq (4).

$$\begin{array}{c} \frac{\partial c}{\partial t}=D_{{\text{O}}_{2}}\frac{\partial^{2}c}{\partial z^{2}}-R_{\text{max}}\frac{c}{K_{m}+c} \end{array}$$ (4)

When the concentration of oxygen is large, *c* ≫ *K*_*m*_, the penetration depth of oxygen scales in proportion to the square root of the oxygen concentration at the boundary.

When the oxygen concentration is below *K*_*m*_, the reaction-diffusion equation is reduced to:

$$\begin{array}{c} \frac{\partial c}{\partial t}=D_{{\text{O}}_{2}}\frac{\partial^{2}c}{\partial z^{2}}-\frac{R_{\text{max}}}{K_{m}}c \end{array}$$ (5)

with the steady state solution

$$\begin{array}{c} c\left(z\right)=c_{0}\;{\text{e}}^{-\lambda_{z}z} \end{array}$$ (6)

for thick biofilms.

The first-order assumption gives the relationships between *K*_*m*_ and *R*_max_:

$$\begin{array}{ccc} R_{\text{max}}=K_{m}D_{{\text{O}}_{2}}\lambda_{z}^{2} & & \lambda_{t}=D_{{\text{O}}_{2}}\lambda_{z}^{2} \end{array}$$ (7)

These relationships make it possible to estimate *R*_max_. Although estimates of this parameter were already made in the previous analysis of the respirometry experiments, it would be appropriate to compare the maximum consumption in a biofilm culture with the planktonic cultures used in the respirometry experiments. The fit was made on the tail of the last oxygen profile in Fig 1 such that a steady-state approximation was appropriate, and for data points below 4 *μ*M such that a first-order reaction could be assumed. The fit was done with the Levenberg-Marquardt method in MATLAB R2016b. The result of the interpolation and fit is shown in S5 Fig. Prior to fitting the data were smoothed using cubic spline in interp1 in MATLAB R2016b and an offset correction was included in the fit.

The resulting estimate of *R*_max_ is 81 *μ*M/min in the upper part of the biofilm. This value was calculated by choosing a representative value for *K*_*m*_ of 3.8 *μ*M, the mean of the cutoffs obtained by Arai *et al.* (2014) [23] of the *bo*_3_, CIO, and *aa*_3_ enzymes, which are the ones we expect to dominate the oxygen consumption during HBOT [23]. As noted, the oxidase regulation of the five oxidases is quite complex [39] and may influence the value of *K*_*m*_.

In the experiment reported by Kolpen *et al.* [3], where the supernatant is replaced and contains fresh nutrient as well as NaNO_3_ during the anaerobic phase, the cell density is higher in the top of the biofilm after the three days of anaerobic growth. We may estimate the cell density from the fitted reaction velocity, *R*_max_ = 81 *μ*M/min and the consumption per bacteria, ∼3 amol/s/cell and arrive at 5 ⋅ 10^8^ CFU/mL near the surface of the biofilm. This is significantly higher than the average density in the biofilm in [3] which is in the order of 10^7^ CFU/mL. The elevated value is a consequence of unevenly distributed growth in the biofilm resulting from oxygen during aerobic growth and NaNO_3_ during anaerobic growth, both being supplied from the biofilm interface. Bearing in mind the uneven cell distribution in the experiment, we expect only a qualitative agreement between the model and the data.

The reaction-diffusion equation in Eq (4) was solved with the numerical solver pdepe in MATLAB that uses an implementation by Skeel *et al.* [40]. Since the supernatant appears to be only moderately mixed we adapted a pragmatic approach where the boundary concentrations at the surface of the biofilm are controlled by a polynomial spline fitted to the experimental data. Similarly the initial concentration of oxygen in the biofilm is constructed as a polynomial spline through the first oxygen profile in Fig 1. The algorithm chosen to fit the parameters in Eq (3) was the Levenberg-Marquardt algorithm in MATLAB.

### Ciprofloxacin model with oxygen consumption

The reaction-diffusion model provides a suitable basis for investigating the effect of oxygen when treating PAO1 biofilms with antibiotics. A generic model is presented here, containing mechanisms that introduce oxygen-induced antibiotic killing.

**Fig 3:**
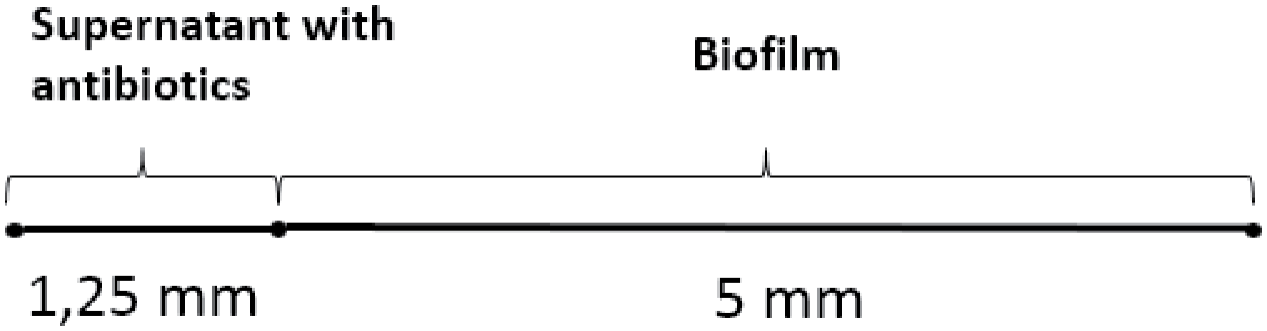
Geometry of ciprofloxacin model coupled with oxygen treatment. Sketch of the 1-dimensional model implemented in MATLAB. The origin is placed at the interface between the supernatant and the biofilm domains, hence the top of the supernatant has coordinate *z* = −1.25 mm, the top of the biofilm has *z* = 0 mm, and the bottom of the biofilm has coordinate *z* = 5 mm.

The geometry of such a model is seen in Fig 3 showing one domain for introduction of antibiotics, i.e. the supernatant and another for the biofilm [3]. The model is intended to provide a qualitative prediction for an experiment in a microtiter plate filled with 5 mm biofilm containing agarose with 1.25 mm well mixed liquid supernatant. Ciprofloxacin and oxygen levels are controlled in the supernatant from where it diffuses into the biofilm. Reflective boundary conditions at the bottom are assumed for oxygen and ciprofloxacin.

Adaptation of bacteria to the antibiotic can be modelled in several ways. However, model 5 from Jacobs *et al.* (2016) [1] fits well in this context as it contains one bacterial population with a reversible adaptation to antibiotics. In order to make the ciprofloxacin killing dependent on the growth rate, we multiply the killing term by the normalised growth rate as seen in the last term in Eq (10). The resulting model is displayed in Eqs (8)–(13), where *z* is the depth into the biofilm.

Inside the biofilm, i. e. for *z* > 0, the model reads

Oxygen:

$$\begin{array}{c} \frac{\partial c}{\partial t}=D_{{\text{O}}_{2}}\frac{\partial^{2}c}{\partial z^{2}}-R_{\text{max}}\frac{c}{K_{m}+c}\;\frac{\varepsilon }{\varepsilon_{\text{norm}}} \end{array}$$ (8)

Ciprofloxacin:

$$\begin{array}{c} \frac{\partial u}{\partial t}=D_{\text{cip}}\frac{\partial^{2}u}{\partial z^{2}} \end{array}$$ (9)

Bacteria:

$$\begin{array}{c} \frac{\partial \varepsilon }{\partial t}=\mu \left(c\right)\left(1-\frac{\varepsilon }{\varepsilon_{\text{max}}}\right)\varepsilon -K_{\text{max}}\;\frac{\mu (c)}{\mu_{\text{max}}}\;\frac{u^{\gamma }}{{\text{KC}}_{50S}^{\gamma }+u^{\gamma }}\varepsilon \end{array}$$ (10)

Adaptation:

$$\begin{array}{c} \frac{\partial \beta }{\partial t}=\left(\frac{S_{\text{max}}\;u}{SC_{50}+u}-\beta \right)k_{\text{out}} \end{array}$$ (11)

Functions:

$$\begin{array}{c} {\text{KC}}_{50S}={\text{KC}}_{50,\text{base}}\;\left(1+\beta \right) \end{array}$$ (12)

$$\begin{array}{c} \mu \left(c\right)=\mu_{\text{max}}\;\frac{c}{K_{m}+c} \end{array}$$ (13)

Eq (8) is the oxygen model from Eq (4) but the reaction term is scaled to be proportional to the fraction of live bacteria in the biofilm. Eq (9) describes the diffusion of ciprofloxacin from the supernatant into the biofilm. Eq (10) describes the change in bacterial concentration within the biofilm and is separated into two terms describing the oxygen-dependent growth and the oxygen-dependent killing effect of ciprofloxacin, respectively. The growth term in Eq (10) is assumed to follow a local logistic growth model with an oxygen dependent specific growth rate following Monod kinetics as shown in Eq (13). This is a fair assumption since the volume fraction occupied by cells is well below 1% in the studied biofilms.

The initial bacterial concentration in the biofilm is set to 5 ⋅ 10^8^ CFU/mL so the simulation captures the estimated oxygen consumption in the top of the biofilm, as observed in the experiments by Kolpen *et al.* [3, 4]. This is about an order of magnitude lower than in a fully developed biofilm. To ensure an oxygen-dependent killing, the killing term is scaled by the normalised oxygen-dependent growth (*μ*(*c*)/*μ*_max_). A similar strategy for incorporating oxygen-dependent killing was used by Stewart (1994) [34] in a model without adaptation. The effect of an adaptive susceptibility to antibiotics has been shown to be considerable [30, 32]. Eq (11) includes both an up and down regulation of adaptation.

The combined oxygen and ciprofloxacin model in Eqs (8)–(13) was solved with pdepe using a 1-dimensional model presented in Fig 3 assuming radial and axial symmetry. We use the estimated oxygen consumption parameters, diffusion parameters, and kinetic parameter values suggested in the literature as detailed in Table 1. Both oxygen and ciprofloxacin are assumed to be well mixed in the supernatant domain and this was implemented in pdepe by setting an artificially high diffusion constant, hence it remains nearly uniformly distributed in the supernatant domain throughout the simulation. As an alternative to using a unit step function to connect the variables across the interface we use a fast shifting logistic function.

**Table 1:** Parameter values used for simulation of the oxygen and ciprofloxacin models.

Parameter	Symbol	Value	Reference
Diffusion constant of oxygen	*D*_O_2__	9.44 mm^2^/h	[41]
Maximum oxygen reaction velocity	*R*_max_	81 *μ*M/min	CS^a^^b^
Oxygen concentration for half-maximum reaction velocity	*K*_*m*_	3.8 *μ*M	[23], CS
Diffusion constant ciprofloxacin	*D*_cip_	1.44mm^2^/h	[42]
Maximum effect of ciprofloxacin	*K*_max_	8 h^−1^	[3], CS^c^
Maximum specific growth rate	*μ*_max_	0.40 h^−1^	[37]
Maximum adaptation	*S*_max_	4	[1]
Initial half-maximum killing rate concentration of ciprofloxacin	*KC*_50,base_	0.2 *μ*g/mL	[2]
Antibiotic concentration that yields 50% of *S*_max_	*SC*_50_	1.06 *μ*g/mL	[1]
Turnover rate constant for adaptation	*k*_*out*_	0.05 h^−1^	[1]
Hill coefficient	*γ*	1.2	[30]
Maximum population density	*ε*_max_	10^9.5^ CFU/mL	[1]
Normalisation constant	*ε*_norm_	5 ⋅ 10^8^ CFU/mL	CS

^a^ Current Study

^b^ Estimated from fit of λ_*t*_ and respirometry experiments.

^c^ Estimated from time-kill curves by Kolpen and coworkers.

The initial conditions in the biofilm are *c*_0_ = 0, *u*_0_ = 0, *ε*_0_ = 10^6^ CFU/mL, and *β*_0_ = 0, and the initial conditions in the supernatant domain are *c*_0_ = the applied oxygen concentration, *u*_0_ = the actual dose, *ε*_0_ = 0 CFU/mL, and *β*_0_ = 0. Hence, the introduction of antibiotics is implemented as a uniform initial condition in the supernatant.

## Results and discussion

### Oxygen model

**Fig 4:**
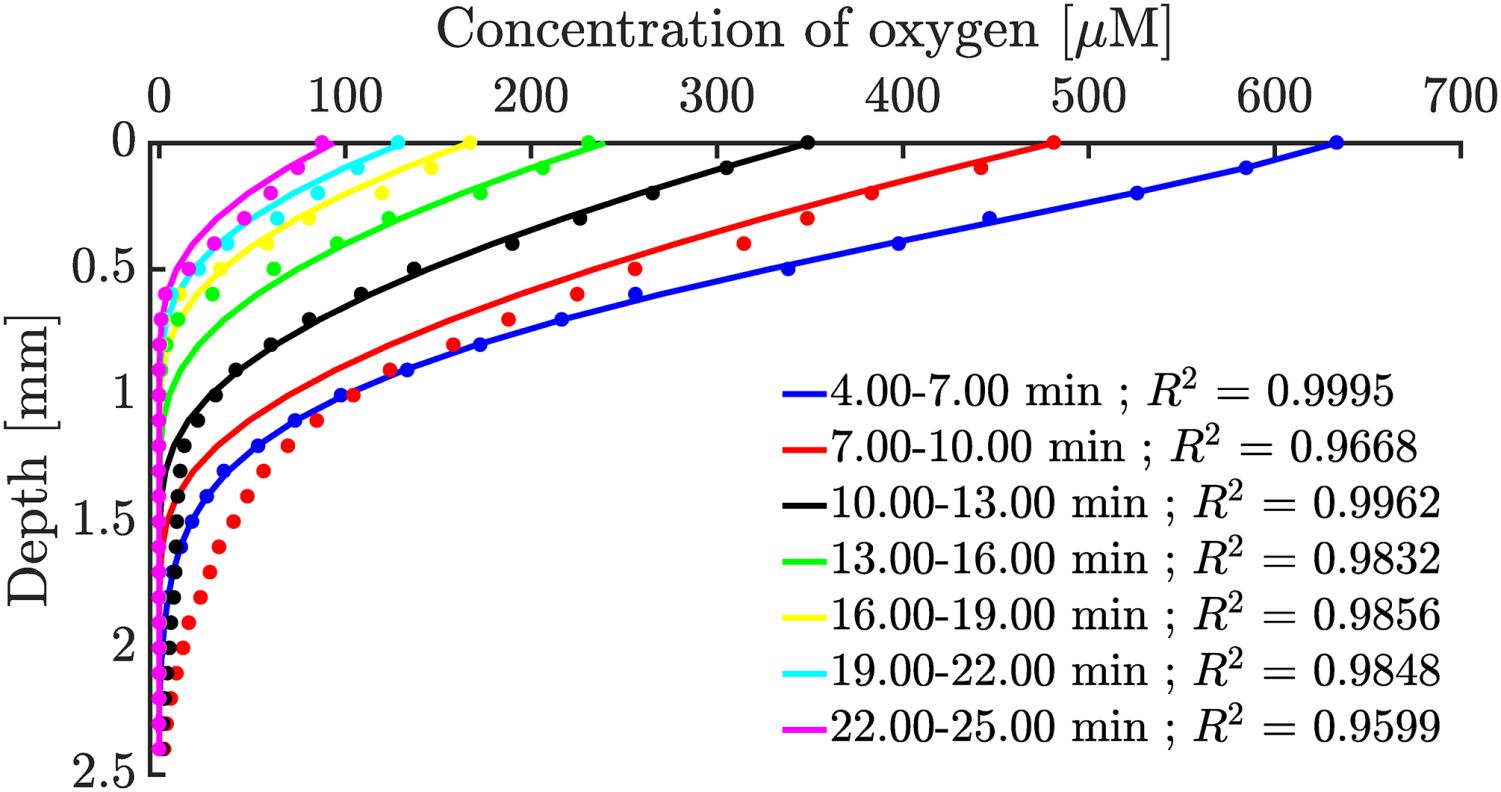
Numerical solutions of the reaction-diffusion equation with a michaelis-menten reaction term. Dots are experimental data from Fig 1 and the coloured lines are matching numerical solutions to Eq (4). The biofilm extends from depth 0 mm to 5 mm, but only the part with measurable oxygen concentration is shown in the figure.

Using the estimated parameters for *R*_max_ and *K*_*m*_, the reaction-diffusion equation, Eq (4), was simulated and compared with the experimental oxygen profiles in Fig 1 as shown in Fig 4.

Looking at Fig 4, the dynamics of the oxygen profiles are qualitatively described by a reaction-diffusion model with a Michaelis-Menten reaction term. As mentioned, *R*_max_ is proportional to the CFU unit count in the biofilm and is thus scaled linearly with the live cell-to-volume fraction in Eq (2). A more detailed model could include a reaction velocity scaled to per bacteria such that the units of *R*_max_ would be in amol⋅s^−1^ bac^−1^. Such a model would be able to describe oxygen consumption across a larger range of bacterial concentrations instead of relying on an estimate of *R*_max_ for a specific cell density as done here. In S1 and S2 Figs it is observed that there is a difference between zero-growth and exponential-growth PAO1 cultures. Generally, the larger *R*_max_ values suggest that exponential-growth cultures are consuming oxygen at a higher capacity per bacterium than zero-growth bacteria. However, further studies are needed to accurately estimate at which bacterial concentration the shift from exponential capacity to stationary capacity happens.

Also, *K*_*m*_ may vary between different experiments depending on their growth conditions prior to starting HBOT because of the five terminal oxidases that respond to different regulatory mechanisms as described by [39].

### Antibiotic model

As mentioned, it has been thoroughly investigated by Kolpen *et al.* [3, 4] that treatment of *P. aeruginosa* with fluoroquinolones like ciprofloxacin shows a time-dependent increased killing when supplementing treatment with high concentrations of oxygen. We have proposed a pharmacodynamic biofilm model accounting for this increased killing, as seen in Eqs (8)–(13).

**Fig 5:**
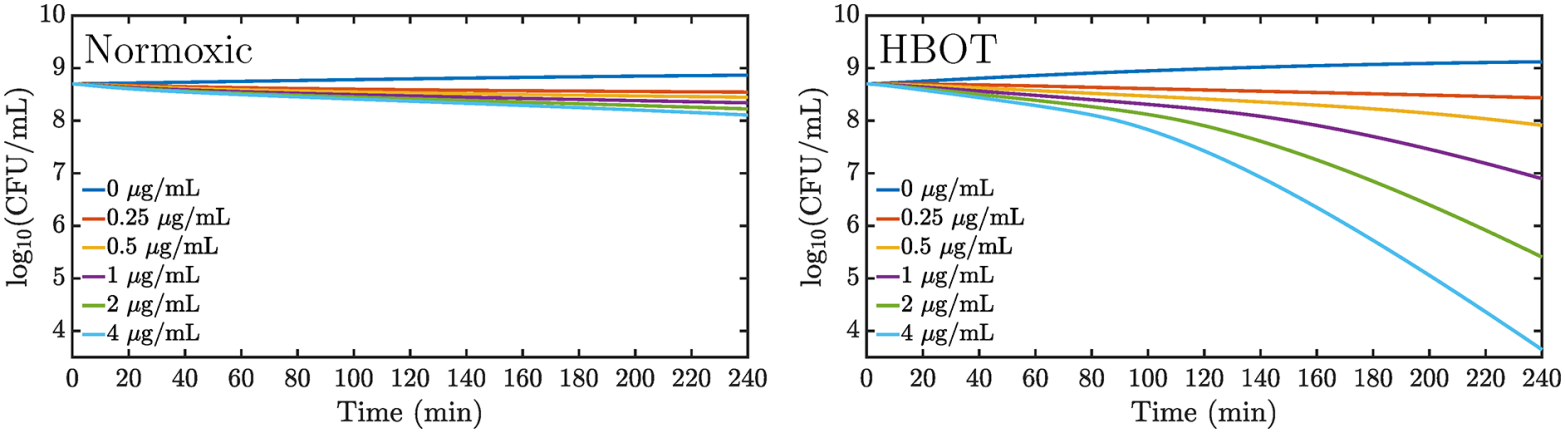
Predicted volume-averaged time kill curves for normoxic oxygen treatment and HBOT. Different dosing schemes over 4 hours of treatment simulated in a 5 mm biofilm model. The initial ciprofloxacin concentrations in the 1.25 mm supernatant are displayed in the figures while the fully equilibrated concentrations are 5 times lower. Left: Normoxic treatment. Right: Hyperbaric oxygen treatment.

The simulations show an oxygen-dependent increased killing as seen in Fig 5 where the volume averaged CFU counts are displayed. At the largest ciprofloxacin dose, we observe 2 orders of magnitude decrease in the CFU after about 2 hours of combined ciprofloxacin and HBOT treatment.

**Fig 6:**
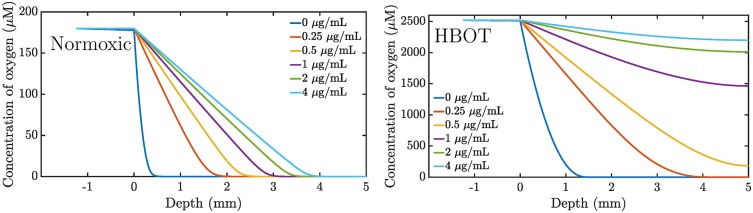
Oxygen penetration. Simulation of oxygen penetration in a 5 mm biofilm following a 4 hour treatment scheme with six different ciprofloxacin dosings. The initial concentration ciprofloxacin in the supernatant is indicated in the figure. The equilibrated concentration is 5 times lower. Left: Normoxic treatment. Right: Hyperbaric oxygen treatment.

**Fig 7:**
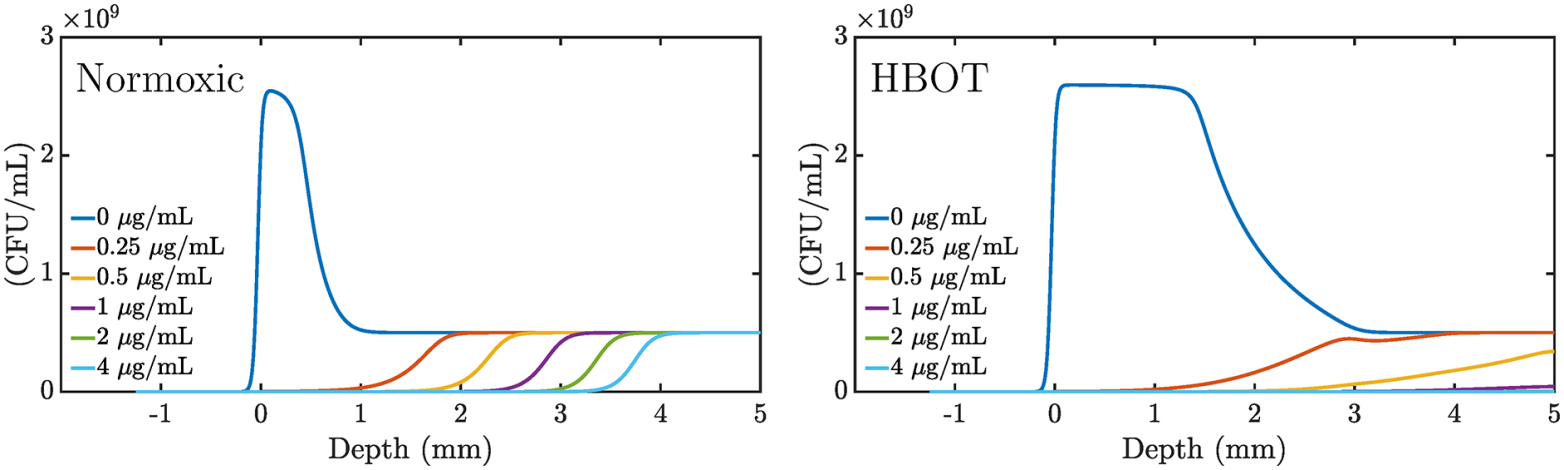
Bacterial killing along the depth dimension of the biofilm. Simulation of a 4 hour treatment scheme with six different doses of ciprofloxacin in a 5 mm biofilm model. The initial concentration ciprofloxacin in the supernatant is indicated in the figure while the equilibrated concentration is 5 times lower. Bacterial density is measured in (CFU/mL). Left: Normoxic treatment. Right: Hyperbaric oxygen treatment.

HBOT increases the penetration depth as seen in Fig 6. This leads to faster growth and metabolic activity, thus making the bacteria more susceptible to antibiotic treatment. However, Fig 5 indicates that the opposing growth is still large enough to compensate the oxygen-dependent killing for initial doses below 1 *μ*g/mL (0.2 *μ*g/mL equilibrated concentration). Fig 7 reveals the distribution of live bacteria in the biofilm: bacteria survive near the bottom and die at the top.

The combined oxygen model with antibiotic killing provides a mechanism for oxygen enhanced killing. The model has a number of opposing effects such as bacterial growth, oxygen-dependent killing, antibiotic adaptation, and oxygen consumption that all contribute to the killing of bacteria. The HBOT makes the culture metabolically active deep into the biofilm. At sufficiently high concentrations of antibiotics the metabolic activity is suppressed and the oxygen is allowed to penetrate even deeper into the biofilm. This, in turn, allows killing even below the normal penetration depth for the oxygen. In this sense, the effect is self-perpetuating. Of course, the parameter values have a significant influence on the simulation and the above dynamics. In further studies it would be interesting to challenge the model against experimental data to see if our model that incorporates oxygen-dependent killing suffices in describing the already observed increased bacterial killing under oxygen treatment by [3, 4].

## Conclusion

A reaction-diffusion model with a Michaelis-Menten reaction term adequately describes *P. aeruginosa*’s oxygen consumption in an artificial PAO1 biofilm. Important model parameters, *K*_*m*_ and *R*_max_, have been determined by performing respirometry experiments and fitting data to an existing PAO1 experiment with HBOT, respectively. Furthermore, we constructed an antibiotic model that included a mechanism for adaptive oxygen-dependent killing of *P. aeruginosa* by introducing a normalised oxygen-growth rate into the antibiotic killing. The increase of the penetration depth of oxygen caused by the HBOT is further boosted by the killing effect from the antibiotics, thus causing the effect to be more efficient than would be naively expected.

In the model, oxygen limitation allows *P. aeruginosa* to survive in biofilms in a dormant state. Hyperbaric oxygen treatment induces metabolic activity and growth, thus increasing the susceptibility for antibiotics leading to a more efficient killing of bacteria. In the specific geometry studied, we find that 4 hours of combined treatment with HBOT and ciprofloxacin at initial concentration 1 *μ*g/mL, results in an equilibrated ciprofloxacin concentration of 0.2 *μ*g/mL and full penetration of oxygen. This, in turn, ensures efficient killing.

The oxygen model presented here describes the oxygen consumption in PAO1 biofilms qualitatively and it is clear that HBOT has the potential to play an important role in the treatment of *P. aeruginosa* biofilm infections with selected antibiotics.

## Supporting information

S1 Fig Fit of the last oxygen profile from Fig 1. Interpolation (black dots) of last oxygen profile (blue dots) from in vitro experiment with PAO1 biofilm after HBOT by Kolpen *et al.* (2016) and subsequent fit of interpolated data (red line).
(EPS)

S2 Fig Oxygen consumption for exponential PAO1 culture. Dots represent experimental data and the red line is the fit. CFU from left to right: 2.2, 2.40, and 2.80 ⋅ 10^7^ mL^−1^.
(EPS)

S3 Fig Oxygen consumption for stationary PAO1 culture. Dots represent experimental data and the red line is the fit. CFU is 1.8 ⋅ 10^9^ mL^−1^ for all three.
(EPS)

S4 Fig Oxygen consumption for stationary PAO1 culture. Dots represent experimental data and the red line is the fit. CFU = 1.7 ⋅ 10^8^ mL^−1^.
(EPS)

S5 Fig Oxygen consumption for stationary PAO1 culture. Dots represent experimental data and the red line is the fit. CFU = 2.3 ⋅ 10^8^ mL^−1^.
(EPS)

S6 Fig Concentration of ciprofloxacin in the supernatant and the biofilm 2, 4, 6, and 8 hours after introduction of 4 *μ*g/mL in the supernatant. (EPS)

## References

[1] Jacobs M, Grégoire N, Couet W, Bulitta JB. Distinguishing Antimicrobial Models with Different Resistance Mechanisms via Population Pharmacodynamic Modeling. PLoS Comput Biol. 2016;12(3):e1004782 doi: 10.1371/journal.pcbi.1004782 26967893 10.1371/journal.pcbi.1004782 PMC4788427

[2] Grillon A, Schramm F, Kleinberg M, Jehl F. Comparative Activity of Ciprofloxacin, Levofloxacin and Moxifloxacin against Klebsiella pneumoniae, Pseudomonas aeruginosa and Stenotrophomonas maltophilia Assessed by Minimum Inhibitory Concentrations and Time-Kill Studies. PLoS One. 2016;11(6):e0156690 doi: 10.1371/journal.pone.0156690 27257956 10.1371/journal.pone.0156690 PMC4892626

[3] Kolpen M, Lerche CJ, Kragh KN, Sams T, Koren K, Jensen AS, et al Hyperbaric oxygen sensitizes anoxic *Pseudomonas aeruginosa* biofilm to ciprofloxacin. Antimicrobial Agents and Chemotherapy. 2017;61(9):AAC.01024–17. 10.1128/AAC.01024-17 PMC5655102 28874373

[4] Kolpen M, Mousavi N, Sams T, Bjarnsholt T, Ciofu O, Moser C, et al Reinforcement of the bactericidal effect of ciprofloxacin on *Pseudomonas aeruginosa* biofilm by hyperbaric oxygen treatment. International Journal of Antimicrobial Agents. 2016;47(2):163–167. doi: 10.1016/j.ijantimicag.2015.12.005 26774522 10.1016/j.ijantimicag.2015.12.005

[5] Lyczak JB, Cannon CL, Pier GB. Lung infections associated with cystic fibrosis. Clinical microbiology reviews. 2002;15(2):194–222. doi: 10.1128/CMR.15.2.194-222.2002 11932230 10.1128/CMR.15.2.194-222.2002 PMC118069

[6] Kiesewetter S, Macek M, Davis C, Curristin S, Chu CS, Graham C, et al A mutation in CFTR produces different phenotypes depending on chromosomal background. Nature genetics. 1993;5(3):274–278. doi: 10.1038/ng1193-274 7506096 10.1038/ng1193-274

[7] Hoegger MJ, Fischer AJ, McMenimen JD, Ostedgaard LS, Tucker AJ, Awadalla MA, et al Impaired mucus detachment disrupts mucociliary transport in a piglet model of cystic fibrosis. Science. 2014;345(6198):818–822. doi: 10.1126/science.1255825 25124441 10.1126/science.1255825 PMC4346163

[8] Boucher R. New concepts of the pathogenesis of cystic fibrosis lung disease. European Respiratory Journal. 2004;23(1):146–158. doi: 10.1183/09031936.03.00057003 14738247 10.1183/09031936.03.00057003

[9] Davies JC, Alton EW, Bush A. Clinical review: Cystic fibrosis. BMJ: British Medical Journal. 2007;335(7632):1255 doi: 10.1136/bmj.39391.713229.AD 18079549 10.1136/bmj.39391.713229.AD PMC2137053

[10] Høiby N, Ciofu O, Bjarnsholt T. Pseudomonas aeruginosa biofilms in cystic fibrosis. Future microbiology. 2010;5(11):1663–1674. doi: 10.2217/fmb.10.125 21133688 10.2217/fmb.10.125

[11] Bjarnsholt T, Jensen PO, Fiandaca MJ, Pedersen J, Hansen CR, Andersen CB, et al Pseudomonas aeruginosa Biofilms in the Respiratory Tract of Cystic Fibrosis Patients. Pediatric Pulmonology. 2009;44(6):547–558. doi: 10.1002/ppul.21011 19418571 10.1002/ppul.21011

[12] Worlitzsch D, Tarran R, Ulrich M, Schwab U, Cekici A, Meyer KC, et al Effects of reduced mucus oxygen concentration in airway Pseudomonas infections of cystic fibrosis patients. Journal of Clinical Investigation. 2002;109(3):317–325. doi: 10.1172/JCI13870 11827991 10.1172/JCI13870 PMC150856

[13] Kolpen M, Kuhl M, Bjarnsholt T, Moser C, Hansen CR, Liengaard L, et al Nitrous Oxide Production in Sputum from Cystic Fibrosis Patients with Chronic Pseudomonas aeruginosa Lung Infection. Plos One. 2014;9(1):e84353 doi: 10.1371/journal.pone.0084353 24465406 10.1371/journal.pone.0084353 PMC3894955

[14] Cowley ES, Kopf SH, LaRiviere A, Ziebis W, Newman DK. Pediatric Cystic Fibrosis Sputum Can Be Chemically Dynamic, Anoxic, and Extremely Reduced Due to Hydrogen Sulfide Formation. Mbio. 2015;6(4):e00767-15, e00767-15. doi: 10.1128/mBio.00767-15 26220964 10.1128/mBio.00767-15 PMC4551978

[15] Kolpen M, Hansen CR, Bjarnsholt T, Moser C, Christensen LD, van Gennip M, et al Polymorphonuclear leucocytes consume oxygen in sputum from chronic Pseudomonas aeruginosa pneumonia in cystic fibrosis. Thorax. 2010;65(1):57–62. doi: 10.1136/thx.2009.114512 19846469 10.1136/thx.2009.114512

[16] Kolpen M, Bjarnsholt T, Moser C, Hansen CR, Rickelt LF, Kühl M, et al Nitric oxide production by polymorphonuclear leucocytes in infected cystic fibrosis sputum consumes oxygen. Clinical and Experimental Immunology. 2014;177(1):310–319. doi: 10.1111/cei.12318 24611476 10.1111/cei.12318 PMC4089181

[17] Moser C, Pedersen HT, Lerche CJ, Kolpen M, Line L, Thomsen K, et al Biofilms and host response—helpful or harmful. Apmis. 2017;125(4):320–338. doi: 10.1111/apm.12674 28407429 10.1111/apm.12674

[18] Jensen PØ, Kolpen M, Kragh KN, Kühl M. Microenvironmental characteristics and physiology of biofilms in chronic infections of CF patients are strongly affected by the host immune response. Apmis. 2017;125(4):276–288. doi: 10.1111/apm.12668 28407427 10.1111/apm.12668

[19] Kragh KN, Alhede M, Jensen PO, Moser C, Scheike T, Jacobsen CS, et al Polymorphonuclear Leukocytes Restrict Growth of Pseudomonas aeruginosa in the Lungs of Cystic Fibrosis Patients. Infection and Immunity. 2014;82(11):4477–4486. doi: 10.1128/IAI.01969-14 25114118 10.1128/IAI.01969-14 PMC4249348

[20] Walters MC, Roe F, Bugnicourt A, Franklin MJ, Stewart PS. Contributions of antibiotic penetration, oxygen limitation, and low metabolic activity to tolerance of Pseudomonas aeruginosa biofilms to ciprofloxacin and tobramycin. Antimicrobial Agents and Chemotherapy. 2003;47(1):317–323. doi: 10.1128/AAC.47.1.317-323.2003 12499208 10.1128/AAC.47.1.317-323.2003 PMC148957

[21] Bjarnsholt T, Alhede M, Alhede M, Eickhardt-Sørensen S, Moser C, Kühl M, et al The in vivo biofilm. Trends in Microbiology. 2013;21(9):466–474. doi: 10.1016/j.tim.2013.06.002 23827084 10.1016/j.tim.2013.06.002

[22] Lobritz MA, Belenky P, Porter CBM, Gutierrez A, Yang JH, Schwarz EG, et al Antibiotic efficacy is linked to bacterial cellular respiration. Proceedings of the National Academy of Sciences of the United States of America. 2015;112(27):8173–80. doi: 10.1073/pnas.1509743112 26100898 10.1073/pnas.1509743112 PMC4500273

[23] Arai H, Kawakami T, Osamura T, Hirai T, Sakai Y, Ishii M. Enzymatic Characterization and In Vivo Function of Five Terminal Oxidases in Pseudomonas aeruginosa. Journal of Bacteriology. 2014;196(24):4206–4215. doi: 10.1128/JB.02176-14 25182500 10.1128/JB.02176-14 PMC4248849

[24] Williams HD, Zlosnik JE, Ryall B. Oxygen, cyanide and energy generation in the cystic fibrosis pathogen Pseudomonas aeruginosa. Advances in microbial physiology. 2006;52:1–71. doi: 10.1016/S0065-2911(06)52001-6 10.1016/S0065-2911(06)52001-6 17027370

[25] Line L, Alhede M, Kolpen M, Kuehl M, Ciofu O, Bjarnsholt T, et al Physiological levels of nitrate support anoxic growth by denitrification of Pseudomonas aeruginosa at growth rates reported in cystic fibrosis lungs and sputum. Frontiers in Microbiology. 2014;5:554 doi: 10.3389/fmicb.2014.00554 25386171 10.3389/fmicb.2014.00554 PMC4208399

[26] Fernández-Barat L, Ciofu O, Kragh KN, Pressler T, Johansen U, Motos A, et al Phenotypic shift in Pseudomonas aeruginosa populations from cystic fibrosis lungs after 2-week antipseudomonal treatment. Journal of Cystic Fibrosis. 2017;16(2):222–229. doi: 10.1016/j.jcf.2016.08.005 27651273 10.1016/j.jcf.2016.08.005

[27] Chmiel JF, Aksamit TR, Chotirmall SH, Dasenbrook EC, Elborn JS, LiPuma JJ, et al Antibiotic management of lung infections in cystic fibrosis. I. The microbiome, methicillin-resistant Staphylococcus aureus, gram-negative bacteria, and multiple infections. Annals of the American Thoracic Society. 2014;11(7):1120–1129. doi: 10.1513/AnnalsATS.201402-050AS 25102221 10.1513/AnnalsATS.201402-050AS PMC5467101

[28] Dwyer DJ, Collins JJ, Walker GC. Unraveling the physiological complexities of antibiotic lethality. Annual review of pharmacology and toxicology. 2015;55:313–332. doi: 10.1146/annurev-pharmtox-010814-124712 25251995 10.1146/annurev-pharmtox-010814-124712

[29] Cao B, Christophersen L, Kolpen M, Jensen PO, Sneppen K, Hoiby N, et al Diffusion Retardation by Binding of Tobramycin in an Alginate Biofilm Model. PLoS ONE. 2016;11(4):e0153616 doi: 10.1371/journal.pone.0153616 27100887 10.1371/journal.pone.0153616 PMC4839563

[30] Grégoire N, Raherison S, Grignon C, Comets E, Marliat M, Ploy MC, et al Semimechanistic pharmacokinetic-pharmacodynamic model with adaptation development for time-kill experiments of ciprofloxacin against *Pseudomonas aeruginosa*. Antimicrobial agents and chemotherapy. 2010;54(6):2379–2384. doi: 10.1128/AAC.01478-08 20368392 10.1128/AAC.01478-08 PMC2876392

[31] Stewart P. Theoretical aspects of antibiotic diffusion into microbial biofilms. Antimicrobial Agents and Chemotherapy. 1996;40(11):2517–2522. 8913456 10.1128/aac.40.11.2517 PMC163567

[32] Tam VH, Schilling AN, Nikolaou M. Modelling time-kill studies to discern the pharmacodynamics of meropenem. Journal of Antimicrobial Chemotherapy. 2005;55(5):699–706. doi: 10.1093/jac/dki086 15772138 10.1093/jac/dki086

[33] Stover C, Pham X, Erwin A, Mizoguchi S, Warrener P, Hickey M, et al Complete genome sequence of Pseudomonas aeruginosa PAO1, an opportunistic pathogen. Nature. 2000;406(6799):959–964. doi: 10.1038/35023079 10984043 10.1038/35023079

[34] Stewart PS. Biofilm accumulation model that predicts antibiotic resistance of Pseudomonas aeruginosa biofilms. Antimicrobial Agents and Chemotherapy. 1994;38(5):1052–1058. doi: 10.1128/AAC.38.5.1052 8067737 10.1128/aac.38.5.1052 PMC188149

[35] Stewart PS, Zhang T, Xu R, Pitts B, Walters MC, Roe F, et al Reaction-diffusion theory explains hypoxia and heterogeneous growth within microbial biofilms associated with chronic infections. Npj Biofilms and Microbiomes. 2016;2(1):16012, 16012. doi: 10.1038/npjbiofilms.2016.12 28721248 10.1038/npjbiofilms.2016.12 PMC5515263

[36] Cunningham A, Visser E, Lewandowski Z, Abrahamson M. Evaluation of a coupled mass transport-biofilm process model using dissolved oxygen microsensors. Water Science and Technology. 1995;32(8):107–114. doi: 10.2166/wst.1995.0274

[37] Roberts ME, Stewart PS. Modeling antibiotic tolerance in biofilms by accounting for nutrient limitation. Antimicrobial Agents and Chemotherapy. 2004;48(1):48–52. doi: 10.1128/AAC.48.1.48-52.2004 14693517 10.1128/AAC.48.1.48-52.2004 PMC310152

[38] Corless RM, Gonnet GH, Hare DE, Jeffrey DJ, Knuth DE. On the LambertW function. Advances in Computational mathematics. 1996;5(1):329–359. doi: 10.1007/BF02124750

[39] Arai H. Regulation and function of versatile aerobic and anaerobic respiratory metabolism in Pseudomonas aeruginosa. Frontiers in microbiology. 2011;2 doi: 10.3389/fmicb.2011.00103 21833336 10.3389/fmicb.2011.00103 PMC3153056

[40] Skeel R, Berzins M. A Method for the Spatial Discretization of Parabolic Equations in one Space Variable. SIAM Journal on Scientific and Statistical Computing. 1990;11(1):1–32. doi: 10.1137/0911001

[41] Han P, Bartels DM. Temperature Dependence of Oxygen Diffusion in H_2_O and D_2_O. Journal of Physical Chemistry. 1996;100(13):5597–5602. doi: 10.1021/jp952903y

[42] Suci P, Mittelman M, Yu F, Geesey G. Investigation of ciprofloxacin penetration into Pseudomonas aeruginosa biofilms. Antimicrobial agents and chemotherapy. 1994;38(9):2125–2133. doi: 10.1128/AAC.38.9.2125 7811031 10.1128/aac.38.9.2125 PMC284696

